# Valorization of donkey milk: Technology, functionality, and future prospects

**DOI:** 10.3168/jdsc.2021-0175

**Published:** 2022-03-03

**Authors:** P. Papademas, P. Mousikos, M. Aspri

**Affiliations:** Department of Agricultural Sciences, Biotechnology and Food Science, Cyprus University of Technology, Limassol, Cyprus 3036

## Abstract

Donkey milk has been in the spotlight for the past 2 decades, mainly because of its potential as a functional food that has positive effects on human health. Nevertheless, challenges remain regarding farming practices, milk yield and milk processing, the introduction of minimal technology, and the use of donkey milk to produce dairy products. In this review, we highlight the fact that interdisciplinary research is needed to provide the scientific community with new knowledge on donkey milk, especially through human clinical trials.

## Highlights

• Donkey milk has a unique chemical composition with a close resemblance to human milk.
• Its processing should be carefully considered to preserve its functional constituents.
• It is considered a high-value, functional product suitable for consumption by special population groups.

## Graphical Abstract

**Figure:**
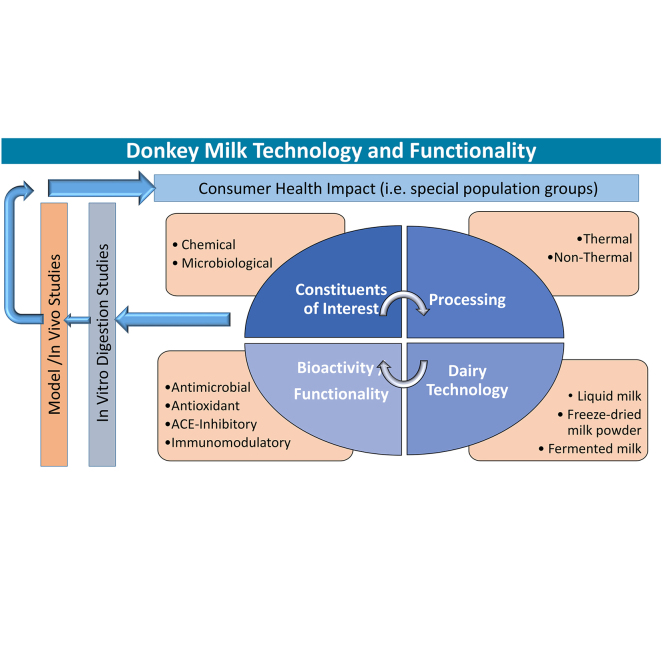
**Summary:** The proposed valorization strategy of donkey milk consists of choosing which milk constituents need to be preserved during processing (e.g., lysozyme, fatty acids) and deciding on the type of processing (i.e., thermal or nonthermal) that will minimize the detrimental changes to milk constituents. By considering factors such as shelf-life, raw milk availability/price, organoleptic characteristics and target consumer groups several dairy products could be manufactured. Donkey milk is considered to has potential therapeutic uses; therefore, its functional or bioactive qualities (e.g., immunomodulation) should be determined by in vitro models and in vivo studies to demonstrate its health benefits in special population groups (e.g., infants, the elderly, and immunocompromised individuals).

Donkey milk was considered by Hippocrates (fourth century bce) and Pliny the Elder (first century ce) as a remedy for different diseases, including liver disease, ulcerations, and asthma (Mansueto et al., 2013). It was used in French orphanages in the 19th century with positive results in terms of infant growth and lowering mortality compared with cow milk consumption (Fantuz et al., 2016). Donkey milk is considered a good alternative to human milk, especially for infants with cow milk protein allergies, due to its particular chemical composition (nutrients and bioactive compounds), palatability, and clinical tolerability (Aspri et al., 2017a). Because of bioactive compounds in donkey milk (i.e., fatty acids, whey proteins, lysozyme, immunoglobulins) and its potential antimicrobial, antioxidant, antidiabetic, anti-inflammatory, and immunomodulatory activities, it should be consumed by specific groups (e.g., the elderly, infants, the immunocompromised).

The global donkey population was estimated at approximately 50.5 million head in 2019, with the majority in Africa (60.6%), Asia (26.2%), and Central/South America (12%) (FAOSTAT, 2021). In developed countries, the donkey population is mainly represented in mountainous and marginal areas where dairy donkey farming is increasingly considered a profitable activity in both food and nonfood sectors (e.g., cosmetology) and is part of the sustainable development of rural areas of many Mediterranean countries (e.g., Italy, Greece, Cyprus; Papademas et al., 2022)

Valorization technologies for donkey milk should be designed to produce safe dairy products by increasing shelf life while preserving or enhancing the functional properties. For example, fermentation of donkey milk by selected characterized indigenous microflora or by commercial probiotic bacteria could enhance the nutritional value of donkey milk and prolong its shelf life (Aspri et al., 2017a). Additionally, nonthermal processing methods developed previously could be used to preserve the biological activity of key milk constituents in donkey milk. This review aims to provide an overview of current knowledge on donkey milk chemical composition, microbiology, processing technologies, dairy products and potential health benefits to a promising niche market, while discussing future research paths.

The donkey mammary gland has a small volume (2–2.5 L), and milk is mostly alveolar rather than cisternal; hence, lactating donkeys need to be milked many times per day after foal separation (i.e., 2–3 h before milking). Both manual and mechanical milking require skill, but donkeys adapt quickly. Milk yield in the donkey is reported to be 3 to 12 mL/kg of BW. De Palo (2021) recently highlighted the need to improve milk yield, milk quality, and farm management practices for efficient donkey milk production. Papademas et al. (2022) reported that donkey milk is white, “thin,” with a lightly sweet, pleasant taste and a pleasant milky aroma, with no persistent aftertaste.

As shown in Figure 1, the content of total protein of donkey milk is low (1.3–1.8 g/100 g) compared with that of cow milk (3.1–3.8 g/100 g) and is closer to that of human milk (0.9–1.7 g/100 g). The protein fraction is rich in whey proteins, which represent 35 to 50% of the nitrogen fraction, whereas whey proteins represent only 20% in cow milk (Aspri et al., 2017a). The individual caseins in donkey milk are (in decreasing order): β-CN (54.3% of total caseins) > α_S1_-CN (35.6%) > α_S2_-CN (7.19%) > κ-CN (2.79%) (see Figure 2). The major whey proteins, as determined by Vincenzetti et al. (2008), are α-LA (1.8 mg/mL), β-LG (3.75 mg/mL), and lysozyme (1.00 mg/mL). Immunoglobulins (11.5%), BSA (6.2%), and lactoferrin (4.5%) are also present in considerable amounts (Guo et al., 2007).

**Figure 1:**
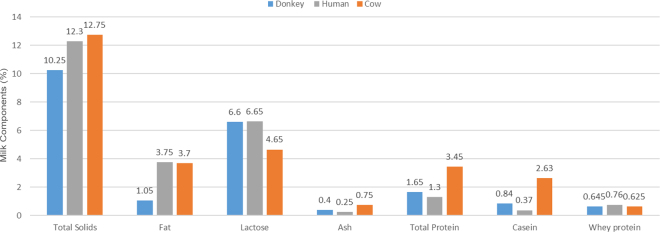
The chemical composition of different milk types. Figure created from data in Aspri et al. (2017b) and Fantuz et al. (2016).

**Figure 2:**
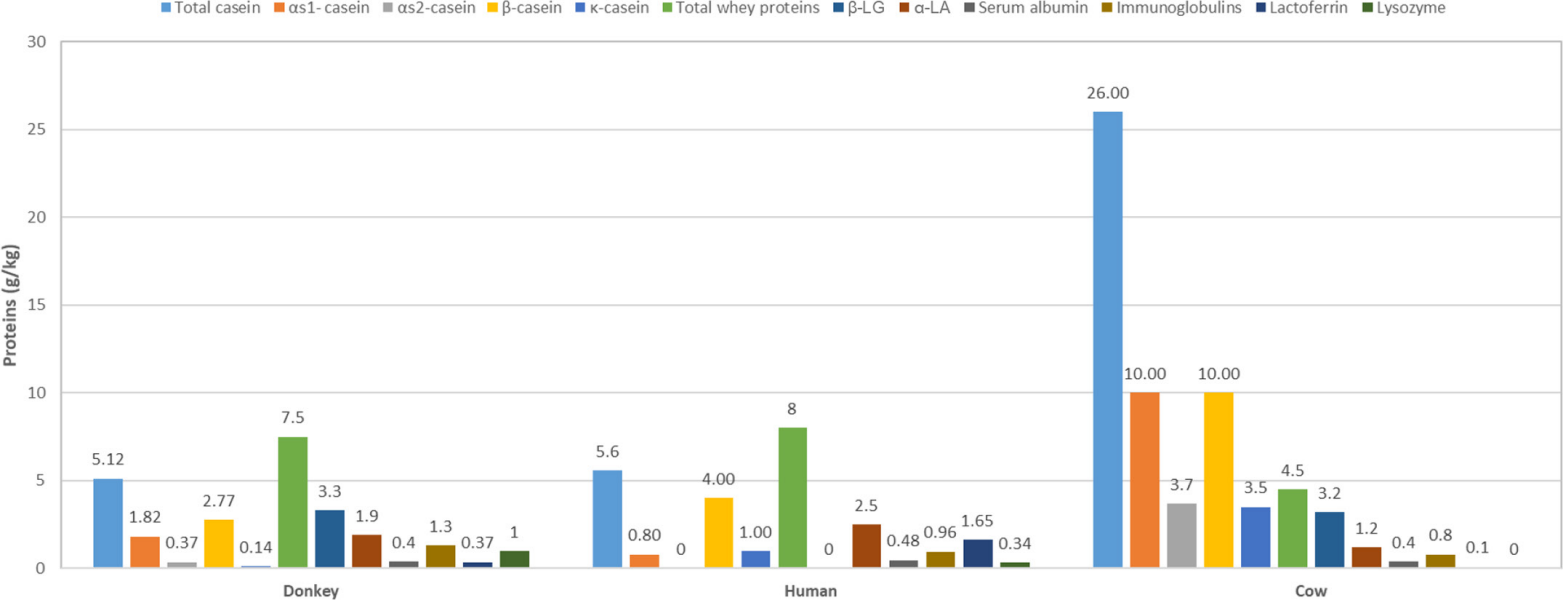
The protein content and their fractions of different milk types. Figure created from data in Aspri et al. (2017b) and Fantuz et al. (2016).

Although data are limited, the amino acid composition of donkey milk protein includes high levels of serine, glutamate, arginine, and valine and much less cysteine, whereas most essential amino acids (isoleucine, leucine, lysine, methionine, phenylalanine, threonine, tyrosine, valine) are also higher than in cow milk (Fantuz et al., 2016; Aspri et al., 2017a).

The fat content of donkey milk ranges from 0.28 to 1.82%, which is lower than that of human and cow milk. The mean gross energy value of donkey milk is 1,748.5 kJ/kg (Martemucci and D'Alessandro, 2012). The low energy value could be a limiting factor for infant nutrition, especially in exclusive diets; hence, donkey milk may be modified by the addition of medium-chain triglycerides or sunflower oil, which alleviates this problem (Carroccio et al., 2000). The lipid fraction is comparable to that of human milk and characterized by high levels of essential fatty acids and α-linolenic acid (C18:3 n-3; ALA) and low SFA (Gastaldi et al., 2010). Moreover, donkey milk is rich in PUFAs, which also predominate in human milk (15–20%), with a high percentage of linoleic acid (LA), a low n-6:n-3 ratio (LA:ALA ratio), and a high ratio of UFA:SFA (Salimei and Fantuz, 2013).

Lactose is the main carbohydrate in donkey milk and its concentration ranges from 6 to 7% (Figure 1). Lactose is responsible for the sweet taste, which is much more pleasing to the palate of the newborn than other breast milk substitutes; moreover, lactose promotes intestinal absorption of calcium that is essential for bone mineralization in infants (Iacono et al., 1992).

Donkey milk is rich in vitamins C (57 mg/L) and D (23 mg/L); levels of vitamins A (17 mg/L) and E (52 mg/L) are much lower than that of cow and human milks (Fantuz et al., 2016). Furthermore, donkey milk contains 410 mg/L thiamine, 640 mg/L riboflavin, 740 mg/L niacin, 1.6 mg/L folic acid, 18.75 μ*M* nicotinic acid, and 5.38 μ*M* vitamin B_6_ (Fantuz et al., 2016). It has been reported that donkey milk contains a high quantity of nicotinic acid, known for its lipid-lowering effect, and moderate amounts of vitamin B_6_ and folic acid, the latter being very important for children's growth (Vincenzetti et al., 2020).

The average ash content in donkey milk is 0.36%, which is slightly higher than that of human milk (about 0.22%) and lower than that of cow milk (0.7–0.8%; Figure 1). The concentrations of Ca (753 mg/L), P (607 mg/L), and Mg (82 mg/L) in donkey milk are intermediate between higher and lower values reported for cow and human milks, respectively, whereas those of K (707 mg/L), Na (180 mg/L), and Cl (337 mg/L) are much lower than in cow milk and closer to those in human milk (Fantuz et al., 2016). Regarding the concentration of microminerals, donkey milk contains similar concentrations of Zn (2,360 mg/L), Mn (20 mg/L), Co (0.5 mg/L), and I (75 mg/L) compared with human milk, whereas Cu (115 mg/L) content is lower (Fantuz et al., 2016).

Milk is an ideal medium for the growth of many microorganisms, including pathogens, spoilage bacteria, and lactic acid bacteria (**LAB**). Microbiological data using cultured-based methods show that raw donkey milk has low total bacterial counts with a mean value of 2.4 to 5.9 log cfu/mL (Papademas et al., 2022). The lower total bacteria count could be attributed to health status, the udder's excellent natural anatomical position and smaller size, which limits bacterial contamination of the teats, and the presence of natural antimicrobial components such as lysozyme, immunoglobulins, lactoferrin, and lactoperoxidase (Salimei and Fantuz, 2013). According to the literature, the most commonly isolated spoilage bacteria are gram-negative bacteria belonging to the *Enterobacteriaceae* family, coliforms, and *Pseudomonas* spp. (Papademas et al., 2021). The presence of food-borne pathogens in raw milk and dairy products is a concern of the dairy industry because these bacteria are associated with human illness. Raw donkey milk has a diverse microbiota, which could include bacteria that cause food-borne illnesses in humans, such as *Cronobacter sakazakii*, *Staphylococcus aureus*, *Bacillus cereus*, *Campylobacter* spp., *Clostridium* spp., *Escherichia coli* O157, *Streptococcus equi*, *Streptococcus equisimilis*, and *Listeria monocytogenes* (Cavallarin et al., 2015; Colavita et al., 2016; Mottola et al., 2018). Interestingly, *Salmonella* spp. and *Mycobacterium* spp. have not yet been reported in raw donkey milk. There is a need to improve handling and hygiene practices during milking and storage of equipment on donkey dairy farms.

In addition to traditional microbiological methods, next-generation high-throughput sequencing (**HTS**) has recently been used to identify bacterial communities present in donkey milk (Luoyizha et al., 2020). According to 16S rDNA sequencing, the bacterial communities of donkey milk consist mostly of members of the phyla *Proteobacteria*, *Firmicutes*, *Bacteroidetes*, *Actinobacteria*, *Acidobacteria*, *Cyanobacteria*, and *Verrucomicrobia* in varying concentrations indicating that the donkey milk microbiota could be strictly related to the different breeding conditions. The most prevalent genera of donkey milk microbiota are gram-negative psychrotrophic bacteria, such as *Pseudomonas* spp., which is consistent with previous studies using cultured-based methods (Cavallarin et al., 2015; Giacometti et al., 2016). *Pseudomonas* spp. are thought to limit the shelf life of donkey milk and have important effects on milk flavor due to their high lipase and protease activities. Other gram-negative bacteria in donkey milk belong to the genera *Ralstonia*, *Cupriavidus*, *Acinetobacter*, *Citrobacter*, *Mesorhizobium*, and *Sphingobacterium*. The high abundance of gram-negative compared with gram-positive bacteria could be due to the presence of lysozyme (Salimei et al., 2004).

The LAB population of donkey milk ranges from 1.0 to 4.2 log cfu/mL, but only a few studies have focused on the isolation and identification of LAB from raw donkey milk. Studies using HTS technology showed a very low relative abundance of the LAB population ranging from 1 to 4.2% of total bacteria (Papademas et al., 2021). Studies on raw donkey milk LAB microbiota showed that it was dominated by *Enterococcus* spp. (*Ent. faecium* and *Ent. faecalis*) and *Streptococcus* spp. (*Streptococcus gallolyticus* ssp. *macedonicus*), which agrees with HTS studies showing a higher percentage of coccus-shaped species compared with bacillus-shaped species (Carminati et al., 2014; Aspri et al., 2017b). Only a small percentage of the LAB population belongs to the *Lactobacillus* and *Leuconostoc* genera. The lactobacilli that have been isolated from donkey milk belong to mesophilic species; that is, *Lacticaseibacillus paracasei*, *Levilactobacillus brevis*, *Ligilactobacillus salivarius*, and *Lactiplantibacillus plantarum* (Papademas et al., 2021).

Thermal treatments are used in the dairy industry to extend milk's shelf life, preserve microbial integrity, and enhance product safety. However, these treatments (depending on the time–temperature profile) can affect nutrient functionality and result in protein denaturation and flavor degradation. The effects of different thermal treatments, such as pasteurization at 63°C for 30 min, high-pressure processing (**HPP**), and their combination, on donkey milk hygiene and microbiological indicators over a 30-d shelf life at 4°C and 12°C were examined by Giacometti et al. (2016). Pasteurization followed by HPP was the most efficient treatment for milk preservation for 30 d. Raw milk treated with HPP had textural defects (e.g., flocculation), making milk unfit for sale. Additionally, the authors demonstrated that pasteurizing raw donkey milk at 63°C for 30 min had no adverse effect on lysozyme content. The effectiveness of a small-scale HTST pasteurizer (72°C/15s) on microbial quality was studied by Giribaldi et al. (2017), indicating that this technology drastically reduced the bacterial population while maintaining lysozyme activity compared with raw donkey milk. Likewise, Ozturkoglu-Budak (2018) investigated the effect of different thermal treatments on the stability of the major whey proteins (lactoferrin, lysozyme, and immunoglobulins); results showed that low temperature treatment and freezing had no significant effect on whey proteins in donkey milk.

Yvon et al. (2019) investigated the effects of various heat treatments on lysozyme activity and microbiological quality of donkey milk. Thermal treatments (72°C for 0.5–3 min) had no effect on lysozyme activity, whereas the most significant decrease in lysozyme activity was reported to be 80°C/10 min and 72°C/8 min, with reductions of 60.79 ± 11.65% and 27.15 ± 5.32%, respectively, compared with raw donkey milk. Miao et al. (2020) examined the effect of heating or ultrasonic treatments on processing stability and evaluated the effect of fermentation on donkey milk rheology and peptide production. The results indicated that donkey milk was more stable when subjected to a lower level of thermal or ultrasonic treatment (i.e., lower surface hydrophobicity, lower centrifugal precipitation rate). In addition, Matera et al. (2022) used a continuous low flow rate pasteurization plant (62.5°C/30 min; 60 dm^3^/h) to evaluate its effect on donkey milk antioxidant capacity and contents of B vitamins, β-LG, and lysozyme. Preliminary results showed that vitamins were retained, whereas the amounts of lysozyme (20–60%) and β-LG (2–22%) were reduced.

Recently, nonthermal treatments, ultra-high-pressure processing (**UHPH**), and UV treatment (UV-C) have gained interest as potential alternatives to traditional thermal processing (Picart-Palmade et al., 2019). A study by Addo and Ferragut (2015) investigated the effect of UHPH on physical, functional, and microbiological characteristics of raw donkey milk compared with pasteurized donkey milk. The authors demonstrated that UHPH treatment resulted in microbial reduction comparable to or better than pasteurization (28-d shelf life), retaining lysozyme activity at a level comparable to that of pasteurized milk during the storage period.

Papademas et al. (2020) studied UV-C treatment (250–260 nm) as a promising nonthermal approach for the inactivation of raw donkey milk microflora, as is optimal for inactivating microorganisms and viruses. The study showed that UV-C could be an alternative to pasteurization for reducing the bacterial population to acceptable levels. Nevertheless, the authors concluded that additional research is needed to determine the effect of UV-C on milk bioactive components.

Freeze-drying can be used for donkey milk preservation as it is a low-temperature process; the process removes moisture, turning milk into powder while preserving the heat-sensitive nutrients. It is a costly method and it should be used only for added-value products.

Processing donkey milk into dairy products (i.e., cheese, yogurt) is challenging, mainly because of the low percentage of α_S_- and κ-caseins and total solids, parameters that affect the coagulum and texture of the end products. Cheese production is very challenging due to the lack of firm curd after renneting (Faccia et al., 2018), which has led to the addition of microbial transglutaminase (MTGase) to donkey milk. Specifically, D'Alessandro et al. (2019, 2021) concluded that MTGase may be used in cheese production from donkey milk to improve curd firmness; chemical composition and cheese color were not significantly affected.

In contrast, the weak coagulum produced during acidification can be used to make yogurt-like products (i.e., drinking dairy products) with probiotic and functional characteristics. The best-known traditional fermented product made from donkey milk that is commercially available (primarily in Kazakhstan, Mongolia, and Russia) is koumiss, which is consumed for its functional and nutritional properties. Traditionally, koumiss was produced by spontaneous fermentation (LAB and yeasts) using raw milk fermented in a leather sack called a “turdusk.” The microbial population is dominated by *Lactobacillus* spp., *Lactococcus* spp., and *Streptococcus* spp. bacteria with the yeasts *Saccharomyces* and *Candida* (Papademas et al., 2022).

Carminati et al. (2014) stated that a high lactose content promoted the growth of probiotic cultures, whereas a high lysozyme content had no negative effect on their growth or acidification capability as starter cultures. A fermented donkey milk beverage developed by Turchi et al. (2017) using a coculture of *Streptococcus thermophilus* and *Lactiplantibacillus plantarum* remained viable for 35 d of refrigerated storage. Perna et al. (2019) investigated the antioxidant activity and phenolic content of donkey kefir enhanced with sulla honey and rosemary essential oil. The authors demonstrated that added polyphenols and the development of polyphenol–protein complexes had a significant positive effect on the antioxidant activity of donkey kefir. Cavalcanti et al. (2021) evaluated the nutritional value of fermented and raw donkey milk, reporting differences in amino acid and fatty acid profiles between the 2 milk types. The authors concluded that both types of milk had significant nutritional value; the fermented milk type had a high viable count of LAB at 6°C for 21 d. Salgado et al. (2021) evaluated the effect of fiber on the viscosity and consumer acceptance of donkey milk yogurt compared with cow milk yogurt, prepared by yogurt cultures. They highlighted that donkey milk yogurts had a higher LAB population in all treatments compared with cow milk yogurts.

The reduced allergenicity of donkey milk (compared with other milk types) has been investigated, and tolerability has been high in most cases. Therefore, donkey milk is a candidate for supplementing breast milk; if adequately supplemented in fat and energy, donkey milk can be successfully used for children suffering from IgE- and non-IgE-mediated cow milk protein allergy. Data showed an adequate increase in BW, length, stature, and body mass index of children after consumption of donkey milk for several months. However, inclusion of donkey milk in the diet of sensitive consumers should be supervised by a nutritionist because of the reported potential for donkey milk proteins to cross-react with cow milk proteins (Mansueto et al., 2013; Martini et al., 2021).

Two similar studies by Coscia et al. (2018) and Bertino et al. (2019) used randomized, controlled clinical trials to compare the feeding tolerance of preterm or very low birth weight newborns to the use of donkey milk–derived fortifier with commercial cow milk–derived fortifier. The authors concluded that donkey milk–derived fortifier improves feeding tolerance compared with standard cow-derived fortifiers, with a similar auxological outcomes. Additionally, Cresi et al. (2020) studied the effects of using donkey milk–derived fortifier compared with cow milk–derived fortifier on gastroesophageal reflux in very low birth weight infants. They concluded that donkey milk–derived fortifier reduced the frequency of gastroesophageal reflux and consequently can be recommended in infants with feeding intolerances.

Because donkey milk has numerous components that could have a bioactive or functional effect, its protein fraction and, more specifically, its soluble protein fraction has attracted research in a quest to provide answers for its bioactive qualities (i.e., antimicrobial, antioxidant, immune-modulation, antihypertensive). Spada et al. (2021) revealed through proteomics that in combination with lysozyme, lactoferrin, and immunoglobulins, the presence of l-amino acid oxidase provides the molecular basis of the antimicrobial potential of donkey milk. Zhang et al. (2021) used proteomics to study the milk fat globule membrane proteins and revealed that most differentially expressed milk fat globule membrane proteins participated in immune system process regulation and lymphocyte activation.

Aspri et al. (2018) has studied the proteomic profile of fermented donkey milk with indigenous isolated microflora (i.e., *Enterococcus faecium*, *Lactobacillus casei*) before and after simulated in vitro gastrointestinal digestion; an increased number of peptides with antihypertensive (angiotensin-converting enzyme inhibitory activity), antimicrobial, and antioxidant activities were present after simulated in vitro gastrointestinal digestion, enhancing potential functionality.

The immunomodulatory effects of donkey milk have been demonstrated by an increase of cytokines involved in the regulation of innate immunity and the onset of local acute inflammatory response—IL-1, IL-6, and tumor necrosis factor-α—both in vitro and in vivo. It is believed that whey proteins, lysozyme, α-lactalbumin, and lactoferrin are involved in immune regulation. Other positive effects are reported on glucose metabolism; for example, where lactose, whey proteins, and bioactive peptides in donkey milk are shown to be involved in insulin response to glucose. Animal studies (in mice) have shown that a donkey milk–supplemented diet improved glucose disposal and insulin resistance, leading to reduction of glucose levels and better tolerance to glucose loads. The beneficial effect of donkey milk on lipid metabolism was illustrated when donkey milk–fed animals had significantly lowered blood triglycerides and reduced fat accumulation, findings attributed to the fact that the skeletal muscle mitochondria of donkey milk–fed animals showed increased respiratory capacity and fatty acid oxidation (Martini et al., 2021).

Gut microbiome studies after donkey milk consumption indicate a possible effect on human health. The effect of lysozyme concentration on shaping the milk's microbiome and modulating the intestinal microflora is a point of discussion. The study of fermented donkey milk should focus on “postbiotics”—mixtures of metabolic products secreted by probiotics in cell-free supernatants (e.g., enzymes, secreted proteins, short-chain fatty acids, vitamins, amino acids, peptides, and organic acids)—and “parabiotics” (dead/inactive cells of probiotics) for their reported anti-inflammatory, antiadhesion, antibiofilm, antiviral, immunomodulatory, and other attributes (Papademas et al., 2020).

Serafini (2021) noted the mounting laboratory-based evidence regarding the positive health effects of donkey milk. Over the past 20 yr, 780 research papers have been published on donkey milk but only 24 (3.1%) were clinical studies and 5 (0.6%) were human trials. Thus, well-designed clinical studies are needed to provide concrete proof for any health-related claim.

Future research on donkey milk should focus on (1) the effect that farm management methods and feeding regimens have on milk yield and quality characteristics; (2) minimal processing methods and treatments of donkey milk to preserve valuable milk constituents; (3) determination of potential bioactivity through deep “-omics” analysis of both milk and dairy products (i.e., fermented) to study any health benefits for humans; and (4) exploring the possibility for donkey milk to be widely used for infant nutrition, complementing breast milk.

In conclusion, there is a need for inter-disciplinary research (involving animal and dairy scientists, molecular biologists, clinicians, and nutritionists) to elucidate the mechanistic basis for the health benefits attributed to donkey milk and to help manufacture dairy products that will offer clinically proven health benefits to consumers.
